# Supplementary material for: Socio-Demographic, Lifestyle, and Clinical Characteristics of Early and Later Weight Status in Older Adults: Secondary Analysis of the ASPREE Trial and ALSOP Sub-Study
Source: Geriatrics (Basel). 2023 Jun 29;8(4):71. doi: 10.3390/geriatrics8040071 (PMC10366913; doi:10.3390/geriatrics8040071)
Supplement: Supplementary file 1 [file geriatrics-08-00071-s001.zip › geriatrics-2257714-supplementary.pdf]

Socio-demographic, lifestyle, and clinical characteristics of early and later weight status in older adults: secondary analysis of the ASPREE trial and ALSOP sub-study

Supplementary Materials S1: Figure S1: Flow diagram of study participants included in this analysis

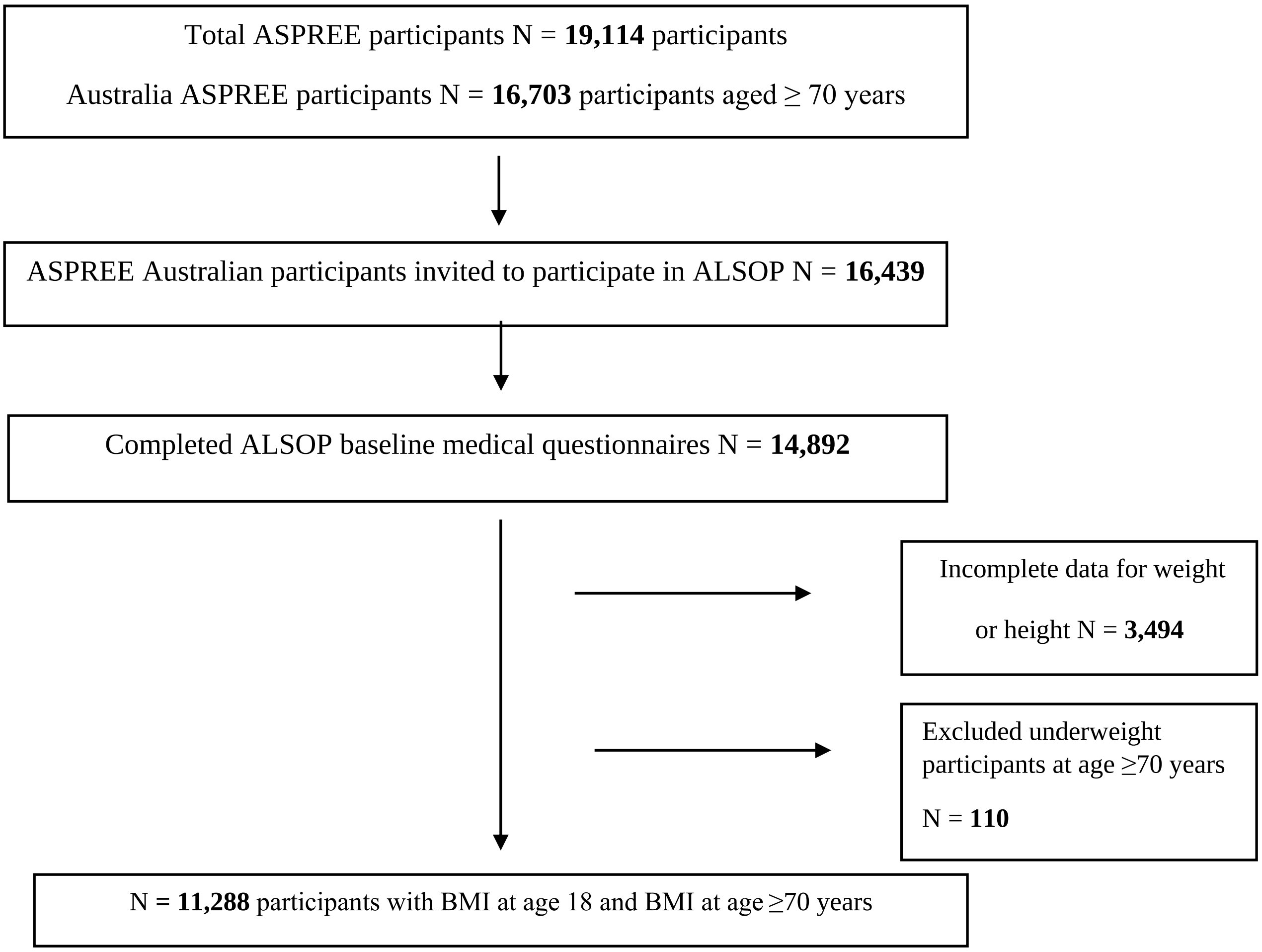

Supplementary Materials S2 Potential Associated Characteristics

Socio-demographic factors included age (years), living situation (living with others, living alone), partner status (partnered, unpartnered), years of education (< 12 years, ≥12 years), residential region (major city, inner region, outer/remote regional) the Socio-Economic Index For Areas (SEIFA, tertiles) [1], paid work (none, full-time/part-time) and volunteer work (not including child minding, baby sitting or caring; no, yes). Lifestyle behaviours included physical activity (no more than light intensity activity in a typical week, moderate or vigorous activity) in middle age and baseline (age ≥70), smoking status (never, former/current); alcohol intake (never, former/current – low risk, current– high risk) defined using Australian guidelines of ≤40g pure ethanol (four standard drinks) on any one day, and ≤100g pure ethanol in a week [2]; and social health (social isolated/low social support/lonely, none). Self-reported health measures included depressive symptoms assessed using the Center for Epidemiologic Studies Depression Scale [3] (CES-D 10: ≥8, <8); and Health Related Quality of Life (HRQoL) assessed using the Medical Outcomes Study 12-item short form (SF-12) (version-2) [4], providing the physical (PCS) and mental (MCS) component scores [5]. Health measured collected during clinical examination included hypertension (high blood pressure measurement of systolic ≥ 140 mmHg or diastolic ≥ 90 mmHg or use of anti-hypertensive medications); diabetes mellitus (self-report of diabetes or fasting glucose ≥ 126 mg/dL or diabetes medications); dyslipidaemia (cholesterol-lowering medications or high serum cholesterol (total ≥212 mg/dL, or High Density Lipoprotein ≥240 mg/dL or Low Density Lipoprotein >160 mg/dL); and cognitive function (global cognition, the Modified Mini-Mental State Examination (3MS) [6], verbal fluency: the Controlled Oral Word Association Test (COWAT) [7], attention and psychomotor speed: the Symbol Digit Modalities Test (SDMT) measured [8], and episodic memory: the delayed recall task from the Hopkins Verbal Learning Test—Revised (HVLT-R)) [6].

1. Australian Bureau of Statistics 2033.0.55.001—Census of Population and Housing: Socio-Economic Indexes for Areas (SEIFA), Australia, 2016: The Index of Relative Socio-Economic Advantage and Disadvantage (IRSAD). Available online: https://www.abs.gov.au/ausstats/abs@.nsf/mf/2033.0.55.001 (accessed on 8 August 2022).
2. Australian Guidelines to Reduce Health Risks from Drinking Alcohol | NHMRC Available online: https://www.nhmrc.gov.au/about-us/publications/australian-guidelines-reduce-health-risks-drinking-alcohol (accessed on 8 August 2022).
3. Radloff, L.S. The CES-D Scale: A Self-Report Depression Scale for Research in the General Population. *Appl. Psychol. Meas.* **1977**, *1*, 385–401, doi:10.1177/014662167700100306.
4. Ware, J.; Kosinski, M.; Keller, S.D. A 12-Item Short-Form Health Survey: Construction of Scales and Preliminary Tests of Reliability and Validity. *Med. Care* **1996**, *34*, 220–233, doi:10.1097/00005650-199603000-00003.
5. User’s Manual for the SF-12v2 Health Survey (with a Supplement Documenting SF-12 Health Survey) – ScienceOpen Available online: https://www.scienceopen.com/document?vid=2afd0468-f217-4cf3-aab7-783e1c9c02f1 (accessed on 8 August 2022).
6. Ryan, J.; Woods, R.L.; Britt, C.; Murray, A.M.; Shah, R.C.; Reid, C.M.; Kirpach, B.; Wolfe, R.S.; Nelson, M.R.; Lockery, J.E.; et al. Normative Performance of Healthy Older Individuals on the Modified Mini-Mental State (3MS) Examination According to Ethno-Racial Group, Gender, Age, and Education Level. *Clin. Neuropsychol.* **2019**, *33*, 779–797, doi:10.1080/13854046.2018.1488996.
7. Nutter-Upham, K.E.; Saykin, A.J.; Rabin, L.A.; Roth, R.M.; Wishart, H.A.; Pare, N.; Flashman, L.A. Verbal Fluency Performance in Amnestic MCI and Older Adults with Cognitive Complaints. *Arch. Clin. Neuropsychol. Off. J. Natl. Acad. Neuropsychol.* **2008**, *23*, 229–241, doi:10.1016/j.acn.2008.01.005.
8. Smith, A. *Symbol Digit Modalities Test*; Western psychological services Los Angeles, 1973;

Supplementary Materials S3: Table S3: BMI at age 18 and at age 70 by early and later life weight status (n=11,288)

	Early and later life weight status*					
**Characteristic**	**Healthy weight**	**Overweight**	**Non-obesity to obesity**	**Obesity to non-obesity**	**Early and later life obesity**	**P value**
**N (%)**	3,494 (30.9%)	5,135 (45.5%)	2,431 (21.6%)	95 (0.8%)	133 (1.2%)	
**Mean ±SD**						
**BMI (kg/m^2) at age 18**	20.8 ±2.1	22.3 ±2.8	23.1 ±3.1	34.2 ±7.1	32.7 ±3.2	0.0001
**BMI (kg/m^2) at age ≥70**	22.8 ±1.5	27.1 ±1.6	33.3 ±3.2	26.4 ±2.8	35.7 ±4.2	0.0001
**N (%)**						
**BMI categories at age 18**						
Underweight	483 (13.8%)	446 (8.7%)	192 (7.9%)	0	0	<0.001
Normal weight	3,011 (86.2%)	3,725 (72.5%)	1,537 (63.2%)	0	0	
Overweight		964 (18.8%)	702 (28.9%)	0	0	
Obese	0	0	0	95 (100%)	133 (100%)	
**BMI categories at age ≥70**						
Underweight	(Excluded)					<0.001
Normal weight	3,494 (100%)	210 (4.1%)	0	26 (27.4%)	0	
Overweight	0	4,925 (95.9%)	0	69 (72.6%)	0	
Obese	0	0	2,431 (100%)	0	133 (100%)	

*Healthy weight (at both age 18 and ≥70y), overweight (at either or both times), obesity (age 18y) to non-obese (age ≥70), non-obese (age 18y) to obesity (age ≥70y), and early and later life obesity (at age 18 and ≥70y).
N=number of observations; SD= standard deviation; p values are from t-test and Wilcoxon rank-sum test (continuous variables) Chi-square tests (categorical variables).
BMI: body mass index; (BMI categories): underweight (<18.5), normal (18.5–25), overweight (25–30), obese (≥30)
